# Serial immunomonitoring of cancer patients receiving combined antagonistic anti-CD40 and chemotherapy reveals consistent and cyclical modulation of T cell and dendritic cell parameters

**DOI:** 10.1186/s12885-017-3403-5

**Published:** 2017-06-15

**Authors:** Alison M. McDonnell, Alistair Cook, Bruce W. S. Robinson, Richard A. Lake, Anna K. Nowak

**Affiliations:** 1 School of Medicine and Pharmacology, The University of Western Australia, Crawley, WA 6009 Australia; 2 National Centre for Asbestos Related Diseases, The University of Western Australia, Crawley, WA 6009 Australia; 3 Department of Medical Oncology, Sir Charles Gairdner Hospital, Nedlands, WA 6009 Australia; 4 Department of Respiratory Medicine, Sir Charles Gairdner Hospital, Nedlands, WA 6009 Australia

**Keywords:** Mesothelioma, Dendritic cells, Prognosis

## Abstract

**Background:**

CD40 signalling can synergise with chemotherapy in preclinical cancer models, and early clinical studies are promising. We set out to define the immunological changes associated with this therapeutic combination to identify biomarkers for a response to the therapy. Here, we present serial immunomonitoring examining dendritic cell and T cell subpopulations over sequential courses of chemoimmunotherapy.

**Methods:**

Fifteen patients with mesothelioma received up to six 21-day cycles of pemetrexed plus cisplatin chemotherapy and anti-CD40 (CP-870,893). Peripheral blood was collected weekly, and analysed by flow cytometry. Longitudinal immunophenotyping data was analysed by linear mixed modelling, allowing for variation between patients. Exploratory analyses testing for any correlation between overall survival and immunophenotyping data were undertaken up to the third cycle of treatment.

**Results:**

Large statistically significant cyclical variations in the proportions of BDCA-1+, BDCA-2+ and BDCA-3+ dendritic cells were observed, although all subsets returned to baseline levels after each cycle and no significant changes were observed between start and end of treatment. Expression levels of CD40 and HLA-DR on dendritic cells were also cyclically modulated, again without significant change between start and end of treatment. CD8 and CD4 T cell populations, along with regulatory T cells, effector T cells, and markers of proliferation and activation, showed similar patterns of statistically significant cyclical modulation in response to therapy without changes between start and end of treatment. Exploratory analysis of endpoints revealed that patients with a higher than average proportion of BDCA-2+ dendritic cells (*p* = 0.010) or a higher than average proportion of activated (ICOS+) CD8 T cells (0.022) in pretreatment blood samples had better overall survival. A higher than average proportion of BDCA-3+ dendritic cells was associated with poorer overall survival at both the second (*p* = 0.008) and third (*p* = 0.014) dose of anti-CD40.

**Conclusions:**

Substantial cyclical variations in DC and T cell populations during sequential cycles of chemoimmunotherapy highlight the critical importance of timing of immunological biomarker assessments in interpretation of results and the value of linear mixed modelling in interpretation of longitudinal change over a full treatment course.

**Trial registration:**

Australia New Zealand Clinical Trials Registry number ACTRN12609000294257 (18th May 2009).

## Background

As a variety of immunotherapies progress toward clinical approval, it is becoming more important to identify biomarkers to assess the clinical activity of these drugs; both to begin to understand what immunobiological changes are induced, and to identify those patients who are likely to benefit from these potentially toxic and often costly treatments. The addition of chemotherapy to immunotherapy in combination treatments is under intense investigation, however there is limited understanding of how concurrent chemotherapy may affect putative biomarkers of immunotherapy, and how to analyse and interpret these in the context of the cyclical changes in immunological parameters induced by cytotoxic treatments. Here, we present further immune biomarker data from a recent chemoimmunotherapy clinical trial conducted in patients with mesothelioma, and discuss the complexity of interpreting this information in the context of prediction and prognosis.

CD40 is a member of the TNF receptor superfamily primarily expressed on antigen-presenting cells (APC), e.g. dendritic cells (DC), B cells and monocytes, but also found on some non-lymphoid cells such as epithelial and endothelial cells, fibroblasts, and some tumours [1]. During the T cell-mediated immune response, CD40 ligand (CD40L; CD154) -expressing CD4 helper T cells can activate APC through CD40 signalling. These APC can in turn provide a ‘licence-to-kill’ signal to CD8 cytotoxic T cells - the main effectors in immune-mediated tumour regression [2].

Extensive preclinical studies of anti-CD40 therapy have shown efficacy in various tumour model systems, and several clinical agents targeting the CD40 signalling axis have been or are currently under investigation [3]. CP-870,893 is a fully human IgG CD40 agonist antibody that has shown promise as a single agent in patients with solid tumours, although overall response rates are still low [4]. Although CP-870,893 infusion was predicted from preclinical studies to induce tumour-specific cell-mediated immune responses, this remains to be fully confirmed in the clinical setting.

Agonistic anti-CD40 can synergise with chemotherapy and cure advanced tumours in mice, especially when administered after chemotherapy [5, 6]. This post-chemotherapy activity of agonistic anti-CD40 is hypothesised to occur by activating DC that have become ‘loaded’ with antigen from chemotherapy-induced tumour cell death, inducing expression of costimulatory molecules CD80 and CD86 and increased production of IL12 amongst other cytokines [7]. CP-870,893 has been investigated in conjunction with chemotherapy in early phase clinical trials, mostly in patients with advanced, treatment-resistant disease [7, 8]. In studies in pre-treated patients, around 20% of participants achieved objective tumour regression. In our recent first-line mesothelioma trial (a phase Ib dose escalation study in combination with chemotherapy), 40% of patients achieved a partial response [9].

Currently identified pharmacodynamic effects of CP-870,893 as a monotherapy are most obvious in the B cell compartment, with depletion and activation of peripheral B-cells occurring within 72 h of infusion [4]. Previous studies have also reported detectable modulation of DC by CP-870,893; in particular, depletion of CD11c^+^CD123^dim^CD14^−^ DC from peripheral blood, and in vitro increases in HLA-DR expression by monocyte-derived DC [10, 11]. Weekly CP-870,893 monotherapy halved circulating lymphocyte concentrations after 48 h before returning to pre-treatment levels; this depletion was not observed when dosing occurred every three weeks [4, 10]. Thus, the pharmacodynamic effects of CP-870,893 are still somewhat undefined – particularly when combined with chemotherapy. Specifically, prolonged immune modulation over the longer term of a full course of treatment has not been characterised.

We recently showed that B-cell depletion and activation also occurs over several cycles of combined CP-870,893 plus pemetrexed and cisplatin chemotherapy in patients with mesothelioma [9]. Here, we present further in-depth flow cytometric analysis of patient peripheral blood mononuclear cells (PBMC) collected longitudinally throughout this study, in order to enhance our understanding of the immunobiology of combination chemoimmunotherapy and the unique challenges and considerations of analysis in this setting. We identify cyclical variations in PBMC subpopulations repeated with each cycle of chemo-immunotherapy, and identify potential relevant biomarkers of clinical activity in dendritic cells, CD8+ effector cells and regulatory T cells in response to anti-CD40 agonist treatment in the context of chemotherapy. We present statistical analysis techniques which may inform other investigators in serial immunomonitoring of chemoimmunotherapy.

## Methods

### Patients

#### Clinical trial designs

The clinical trial was a prospective, single-centre, phase Ib trial of cisplatin and pemetrexed with CP-870,893 [9]. A 3 + 3 phase I design was used with a 6-patient expansion cohort at the maximum tolerated dose (MTD) of CP-870,893. The primary endpoint was the MTD of CP-870,893. Secondary endpoints included toxicity (NCI CTC Version 3.0), objective tumour response as measured by the modified RECIST criteria [12] and by fluorodeoxyglucose positron emission tomography (FDG-PET) [13], time to progression (TTP), and overall survival (OS).

#### Eligibility

Eligibility criteria have been previously described in detail [9]. In brief, all patients had confirmed malignant pleural mesothelioma, Eastern Co-operative Oncology Group (ECOG) performance status (PS) 0–1, and were planned for first-line cisplatin/pemetrexed. Exclusions specific to study drug were: history of venous thromboembolism or severe autoimmunity. The protocol was approved by the Institutional Human Research Ethics Committee and participants provided written informed consent. Australia New Zealand Clinical Trials Registry number ACTRN12609000294257.

#### Treatment and outcome assessment

Patients received cisplatin 75 mg/m2 and pemetrexed 500 mg/m2 on day 1 of a 21 day cycle to maximum 6 cycles with vitamin B12 and folate supplementation. CP-870,893 was given on day 8 each cycle, at three dose levels in consecutive patient cohorts: 0.1 mg/kg; 0.2 mg/kg; with 0.15 mg/kg as a de-escalation level. Patients received premedication for cytokine release reaction before CP-870,893 administration as previously described [9]. Prophylactic medications for chemotherapy included corticosteroids (days −1 to 2) and antiemetics; other anti-cancer treatments were not allowed. Chemotherapy was stopped before 6 cycles in the event of progression, unacceptable toxicity, or patient withdrawal; in this event, CP-870,893 was also stopped. Patients with stable or responding tumour at 6 cycles could continue single agent CP-870,893 every 21 days for a further 6 cycles at the same dose level, ceasing on progression or toxicity. Complete blood count, hepatic and renal function tests, and toxicity assessment were performed weekly on combination treatment and three-weekly on CP-870,893 alone. Clinical, imaging, and time to event outcome assessments have been described previously [9].

### Cell preparation

#### Peripheral blood volumetric cell counts

Whole blood was analysed by flow cytometry on the day of collection to obtain absolute volumetric cell counts (cells per mL) of CD3^+^CD8^+^ and CD3^+^CD4^+^ T cells. Blood samples were stained using CD4-AlexaFluor488, CD3-PE and CD8-PECy7 antibodies as detailed in Table 1. Fixation and red blood cell lysis was performed using BD FACS lysing buffer, and data collected by three-color analysis using a Millipore Guava and Guava ExpressPro Software.

**Table 1:** List of antibodies

Antigen	Fluor	Clone	Isotype	Supplier	Catalogue #	Panel	Dilution
BDCA-1 (CD1c)	PE	AD5-8E7	ms IgG1	Miltenyi	130–090-508	1	1/10
BDCA-2 (CD303)	FITC	AC144	ms IgG1	Miltenyi	130–090-510	1	1/10
BDCA-3 (CD141)	APC	AD5-14H12	ms IgG1	Miltenyi	130–090-907	1	1/10
HLA-DR	V500	G46.6	ms IgG2a	BD Biosciences	561,224	1	1/100
CD3	PerCP-Cy5.5	SK7	ms IgG1	Biolegend	344,808	1	1/100
CD14	PerCP-Cy5.5	HCD14	ms IgG1	Biolegend	325,622	1	1/100
CD16	PerCP-Cy5.5	3G8	ms IgG1	Biolegend	302,028	1	1/100
CD19	PerCP-Cy5.5	HIB19	ms IgG1	Biolegend	2,072,925	1	1/100
CD56	PerCP-Cy5.5	HCD56	ms IgG1	Biolegend	318,322	1	1/100
CD40	APC-H7	5C3	ms IgG1	BD Biosciences	561,211	1	1/10
CD4	AF488	RPA-T4	ms IgG1	BD Biosciences	557,695	2	1/20
CD3	PE	SK7	ms IgG1	BD Biosciences	347,347	2	1/50
CD8	PECy7	RPA-T8	ms IgG1	BD Biosciences	555,368	2	1/50
CD4	APC-H7	RPA-T4	ms IgG1	BD Biosciences	560,158	3	1/40
Foxp3	PE	PCH101	rt IgG2a	eBioscience	12–4776-42	3	1/20
CD25	APC	M-A251	ms IgG1	BD Biosciences	555,434	3	1/5
CD127	PECy7	eBioRDR5	ms IgG1	eBioscience	25–1278-42	3	1/100
Ki67	FITC	B56	ms IgG1	BD Biosciences	556,026	3,4	1/10
ICOS	PerCP-Cy5.5	C398.4A	ha IgG	Biolegend	313,518	3,4	1/80
CD14	V500	M5E2	ms IgG2a	BD Biosciences	561,391	3,4	1/80
CD19	V500	HIB19	ms IgG1	BD Biosciences	561,121	3,4	1/80
CD3	V450	UCHT1	ms IgG1	BD Biosciences	560,365	3,4	1/40
CD8	APC-H7	SK1	ms IgG1	BD Biosciences	560,179	4	1/40
CD38	AF647	HIT2	ms IgG1	Biolegend	303,514	4	1/40
HLA-DR	PECy7	L243	ms IgG2a	BD Biosciences	335,795	4	1/80
Bcl-2	PE	Bcl-2/100	ms IgG1	BD Biosciences	556,535	4	1/10

List of monoclonal antibodies used for flow cytometric staining. Panels were used for dendritic cell staining of PBMC (panel 1), absolute cell counts of whole blood (panel 2), Treg staining of PBMC (panel 3), or CD8 T cell staining of PBMC (panel 4). Abbreviations: AF = AlexaFluor, ms = mouse, rt. = rat, ha = hamster

#### PBMC Isolation

**Fig. 1:**
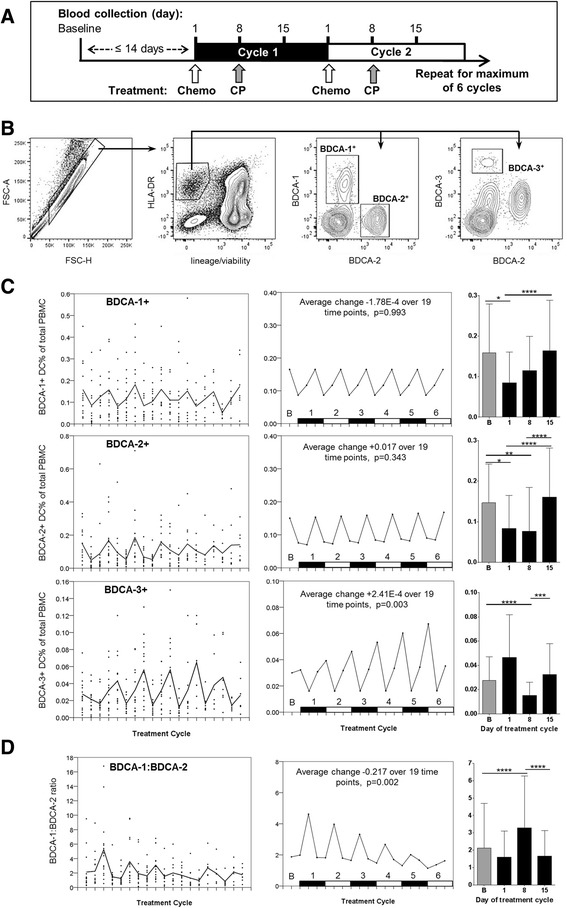
**a** Patient treatment schedule showing timings of study drug administration and PBMC collection; Chemo = pemetrexed/cisplatin chemotherapy, CP = CP-870,893. Blood was collected at baseline, then days 1, 8 and 15 of each treatment cycle for a maximum of 6 cycles combined therapy. **b** Representative flow cytometry data demonstrating gating strategy for DC. Forward scatter (FSC) area vs. FSC-height was used for doublet discrimination. A ‘dump’ channel was used to gate out dead cells (LIVE/DEAD viability stain) plus those staining positively with a CD3/CD14/CD16/CD19/CD56 lineage cocktail (lin^+^). DCs were identified as lin^−^HLA-DR^+^ cells, and respective DC subpopulations identified by BDCA-1, BDCA-2 or BDCA-3. **c** Longitudinal flow cytometry data on DC across six cycles of chemoimmunotherapy, for BDCA-1, BDCA-2 and BDCA-3 subpopulations as a proportion of total PBMC. Left-hand panels show observed values from individual patients, together with their empirical means (solid line), mean and SD at baseline are quoted. Centre panels show results of fitting a linear mixed model; a linear trend over time and additive treatment effects of the day of the treatment yield the corresponding population average curves. Black and white numbered bars on X-axes represent the number of treatment cycles undertaken, time point ‘B’ represent pre-study baseline samples. Y axis scales have been modified from left-hand panels for clarity to show cyclical changes highlighted by modelling. Average change over the duration of the study is described. Right-hand panels show estimated treatment means, showing differences between day 1, day 8, and day 15 of the chemoimmunotherapy treatment over 6 cycles (*P*-values: * <0.05, ** <0.01, *** <0.001, **** < 0.0001). **d** Longitudinal data on the ratio of BDCA-1 to BDCA-2 dendritic cells

Whole blood for PBMC isolation was collected into BD K2EDTA Vacutainers (BD Diagnostics, Australia) weekly during combined treatment (days 1, 8, 15), always prior to treatment administration (Fig. 1a). PBMC were isolated by Ficoll-Paque™ density gradient centrifugation, and cryopreserved in liquid nitrogen until analysis. Dual baseline samples were collected within 14 days of day 1, and pre-treatment on day 1 cycle 1. Serial analyses were performed on cryopreserved PBMCs, with all samples analysed concurrently for individual patients ensuring comparable experimental conditions across time points.

#### PBMC flow cytometry

PBMCs were thawed for 1 min at 37 °C and washed once in RPMI (Invitrogen), followed by two washes in PBS after putting cells into 96-well U-bottom plates (1 × 10^6^ cells / well). PBMC were stained for expression of surface markers using specific anti-human monoclonal antibodies (mAb) comprising three 8-colour panels as detailed in Table 1. Dead cells were identified using LIVE/DEAD Fixable Dead Cell Stain Kit (Thermo Fisher Scientific, Waltham, MA, USA).

DC were identified as staining with MHC Class II (HLA-DR-V500), and negative for a lineage cocktail of CD3-PerCP-Cy5.5, CD14-PerCP-Cy5.5, CD16-PerCP-Cy5.5, CD19-PerCP-Cy5.5, CD56-PerCP-Cy5.5 and LIVE/DEAD Fixable Red viability stain. Within this population, DC subsets were identified by antibodies against BDCA-1-PE, BDCA-2-FITC and BDCA-3-APC. Expression of CD40 was assessed within these subpopulations by CD40-APC-H7 staining.

CD4+ T cells were identified by positive staining for CD3-V450 and CD4-APCH7, and negative for a lineage cocktail of CD19-V500, CD14-V500 and LIVE/DEAD Fixable Aqua viability stain. Tregs were identified within the CD3+ CD4+ T cell population by staining with CD25-APC, Foxp3-PE, and low CD127-PECy7. Within the Treg subset, cells were further assessed for staining by Ki67-FITC and ICOS-PerCP-Cy5.5.

CD8+ T cells were identified by positive staining for CD3-V450 and CD8-APC-H7, and negative for a lineage cocktail of CD19-V500, CD14-V500 and LIVE/DEAD Fixable Aqua viability stain. Within the CD8+ population, activated effector cells were identified by HLA-DR-PECy7 and CD38-AlexaFluor647. These cells were also assessed for staining by Ki67-FITC, ICOS-PerCP-Cy5.5 and Bcl2-PE.

Samples were run on a FACSCanto II flow cytometer using FACSDiva software (both BD Biosciences). At least 50,000 lymphocyte events were collected per sample. Data were analysed using FlowJo software (Tree Star Inc., Ashland, OR, USA).

### Statistical analysis

Linear mixed models were used to analyse the relationship between time and lymphocyte subsets, in addition to testing for interaction. Analysis used the R environment for statistical computing and IBM SPSS for Windows statistical package version 23. Bar graphs showing treatment means were analysed using ANOVA, *P* values are multiplicity adjusted using Dunn multiple comparisons tests.

Exploratory analysis for correlation of DC or T cell subtypes with overall survival (OS) was performed separately for each sample collection time point between baseline and Cycle 3 Day 8. Patients were grouped above or below the median according to proportional presence of the DC or T cell subset under investigation. OS was analysed using the Kaplan Meier method, differences in OS between groups were calculated using the Mantel-Cox Log Rank test. No corrections for multiple comparisons were performed here as analyses were exploratory and hypothesis-generating.

## Results

Sixteen patients with radiologically assessable malignant mesothelioma were enrolled as described previously [9]. Patients were treated and PBMC samples collected as described in the materials and methods section and Fig. 1a. We analysed all patient PBMC samples by flow cytometry and here report on DC, and CD4 or CD8 T cell subsets. Linear mixed modelling of the data allowed us to not only assess immunological changes from week to week in response to different components of the treatment regimen, but also to examine longitudinal changes across 6 cycles of treatment.

### Dendritic cell subpopulations

Blood DC subpopulations were identified by flow cytometry on the basis of BDCA marker expression using the gating strategy as shown in Fig. 1b (see discussion for DC subpopulation characteristics and roles). Whilst our data on the proportion of these DC subpopulations as a fraction of total PBMC showed a wide variability, both within and between individual patients over 6 cycles of chemoimmunotherapy treatment, the pattern of change was cyclical and consistent for BDCA-1^+^ and BDCA-2^+^ (CD303^+^ plasmacytoid) DC (Fig. 1c). In both these DC subsets, a marked proportional decrease of around 50% was observed prior to Day 1 of each cycle, coinciding with pre-chemotherapy medication with the glucocorticoid steroid dexamethasone [14]. The proportion of BDCA-2^+^ DC remained low until Day 15 of each cycle before returning to baseline levels, whereas a recovery in BDCA-1+ DCs was seen a week earlier at Day 8 (Fig. 1c). The less numerous, BDCA-3^+^ (CD141^+^ myeloid), DC showed a more complex profile, with linear mixed modelling indicating a rebound significantly above, baseline levels by Day 1 of each treatment cycle. The ratio of BDCA-1^+^ to BDCA-2^+^ DC was variable, reflecting the differential recovery of BDCA-1^+^ and BDCA-2^+^ subsets on Day 8 and Day 15 respectively, with these differences becoming less pronounced as the number of treatment cycles progressed (Fig. 1d).

### Dendritic cell functional markers

**Fig. 2:**
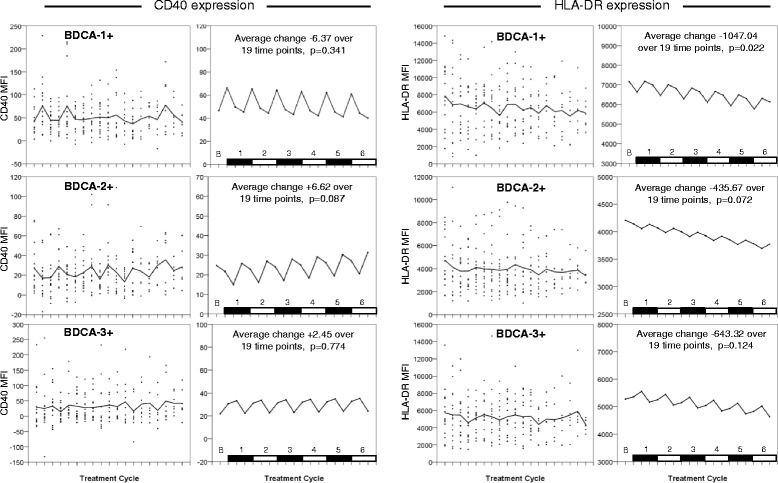
Longitudinal flow cytometry data on DC across six cycles of chemoimmunotherapy, detailing changes in mean fluorescence intensity (MFI) relating to expression levels of CD40 or HLA-DR in BDCA-1, BDCA-2 and BDCA-3 DC subsets. Left-hand panels show observed values from individual patients, together with their empirical means (solid line). Right hand panels show results of fitting a linear mixed model; a linear trend over time and additive treatment effects of the day of the treatment yield the corresponding population average curves. (*P*-values: * <0.05, ** <0.01, *** <0.001, **** < 0.0001)

Relative expression levels of both CD40 and HLA-DR were assessed by mean fluorescence intensity (MFI) of staining (Fig. 2). CD40 was expressed at highest levels on BDCA-1+ DC, and lowest levels on BDCA-3+ DC. Linear mixed modelling revealed a cyclical pattern of CD40 expression on all three subsets. BDCA-1+ DC expressed most CD40 at Day 1 of each cycle (after dexamethasone premedication), returning to baseline (pre-corticosteroid) levels at Day 8 and Day 15. CD40 expression on BDCA-2+ DC was seen to decrease slightly from baseline levels at Day 1, with a more substantial decrease by Day 8 before returning to baseline levels by Day 15 of each cycle. BDCA-3+ DC displayed upregulation of CD40 at Day 1 and Day 8, decreasing back to baseline levels at Day 15. However, none of the DC subsets investigated here exhibited a significant overall change in the levels of CD40 expression across 6 cycles of combined chemoimmunotherapy.

HLA-DR expression was highly variable. Linear mixed modelling indicated that HLA-DR expression levels were also modulated in a cyclical fashion to some degree, and on BDCA-1+ DC showed a statistically significant, but minor, decrease between the start and end of treatment. Cyclical changes in HLA-DR expression on BDCA-2+ DC were minimal, and general HLA-DR levels as measured by MFI were lower than the other DC subsets. HLA-DR expression on BDCA-1+ DC showed a marked decrease at Day 1 of each cycle before rebounding, whereas BDCA-3+ DC displayed an increase in HLA-DR levels at Day 8 followed by a sustained decrease by Day 15 of each cycle.

**Fig. 3:**
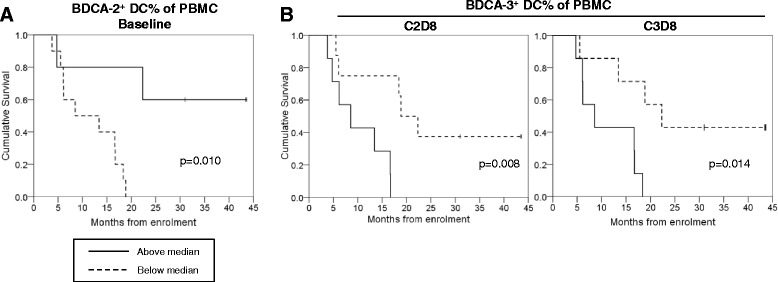
Overall survival analysed by grouping patients above and below the median for (**a**) BDCA-2+ DC % of total PBMC at baseline, and (**b**) BDCA-3+ DC % of total PBMC at cycle 2 day 8 or cycle 3 day 8. Statistical significance calculated using Mantel Cox log rank test. Solid and dotted lines show data above and below the median values, respectively

Flow cytometry data on DC subset representation and relative expression of functional markers underwent exploratory analyses for correlation with overall survival (OS), separately for each time point between baseline and Cycle 3 Day 8. For a full list of parameters analysed, see Table 2. The majority of analyses showed no significant differences in OS above vs below the median, however those patients who had higher than the median proportion of BDCA-2+ DC at baseline have longer OS (Fig. 3a), and patients who have higher than the median proportion of BDCA-3+ DC when CP-870,893 was delivered (Day 8) during Cycle 2 and Cycle 3 had poorer OS (Fig. 3b).

**Table 2:** Data analysed for correlation with overall survival

DC parameters	T cell parameters
DC (HLA-DR+ lin-) % of PBMC	CD8 + % of CD3+
BDCA-1+ DC % of total PBMC	Ki67+% of CD8 T cells
BDCA-2+ DC % of total PBMC	ICOS+% of CD8 T cells
BDCA-3+ DC % of total PBMC	Teff (HLA-DR+CD38+) % of CD8 T cells
BDCA-1+ % of DC	CD3+% of lymphocytes
BDCA-2+ % of DC	CD4+% of CD3+
BDCA-3+ % of DC	Ki67+% of CD4 T cells
BDCA-1+:BDCA-2+ ratio	ICOS+% of CD4 T cells
BDCA-1+:BDCA-3+ ratio	Treg+% of CD4 T cells
BDCA-2+:BDCA-3+ ratio	ki67 + % of Treg (CD25^+^CD127^lo^Foxp3^+^)
BDCA-1+ DC CD40 MFI	ICOS + % of Treg (CD25^+^CD127^lo^Foxp3^+^)
BDCA-1+ DC HLA-DR MFI	
BDCA-2+ DC CD40 MFI	
BDCA-2+ DC HLA-DR MFI	
BDCA-3+ DC CD40 MFI	
BDCA-3+ DC HLA-DR MFI	

List of parameters undergoing exploratory analysis for correlation with OS. Flow cytometry data was used to group patients above or below the median for each parameter, and repeated using data from each sample collection time point from baseline through to Cycle 3 Day 8

### Stability of T cell number and subset distribution

**Fig. 4:**
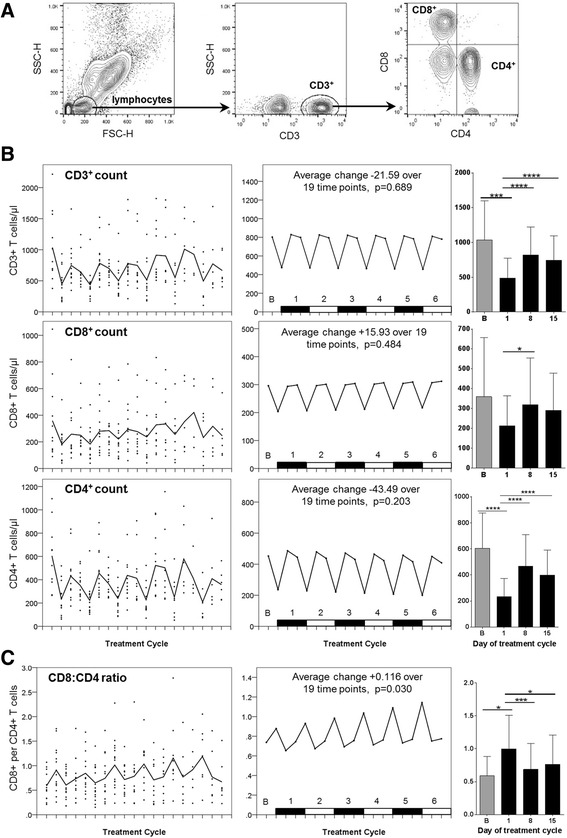
**a** Representative flow cytometry data showing gating strategy on whole blood samples used to obtain absolute volumetric cell count data. Lymphocytes were identified on the basis of FSC vs. SSC, with CD4^+^ or CD8^+^ T cells subsequently identified from within the CD3^+^ subset. **b**-**c** Longitudinal empirical data, linear mixed models and estimated means (left, centre and right-hand panels respectively) for: (**b**) absolute volumetric cell count data; (**c**) ratio of CD8^+^ T cells to CD4^+^ T cells (*P*-values: * <0.05, ** <0.01, *** <0.001, **** < 0.0001)

T cell concentrations per microlitre of peripheral blood were recorded for each sample prior to PBMC isolation (Fig. 4a). Variation in overall number of CD3+ T cells, as well as the proportion of CD4+ and CD8+ cells fluctuated in a repetitive manner each treatment cycle, with the number of T cells halving at Day 1, and returning to baseline levels at Day 8 and Day 15 (Fig. 4b). Treatment did not affect CD4+ and CD8+ numbers in an identical manner, with the CD8+ to CD4+ ratio becoming skewed in favour of CD8+ T cells at Day 1 of each cycle after dexamethasone and immediately prior to chemotherapy, and returning to around baseline levels by Day 8 and Day 15 of each cycle, a pattern consistent with a treatment dependent CD4 depletion. With each additional cycle of chemoimmunotherapy the scale of CD4+ T cell depletion became more pronounced, but remained transient such that the balance between CD4 and CD8 always returned to baseline by Day 15 of each cycle (Fig. 4c).

### CD8+ T cell proliferating, activated and effector populations

**Fig. 5:**
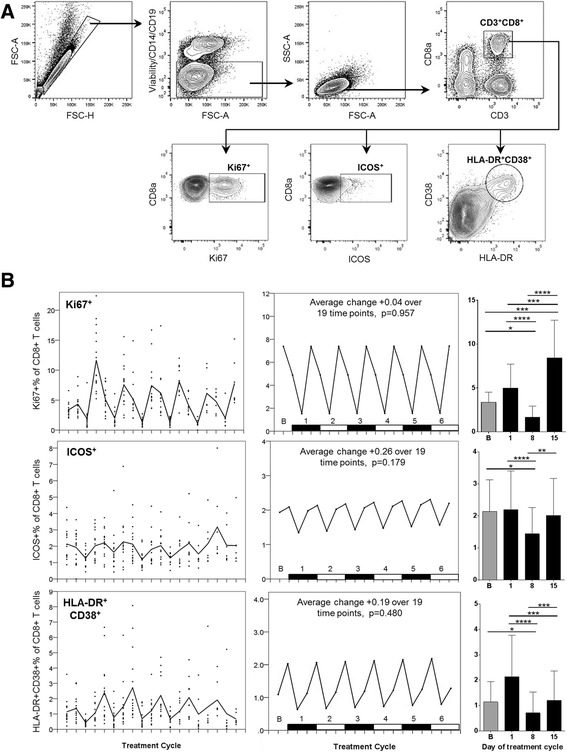
**a** Representative flow cytometry data demonstrating the gating strategy used on PBMC for CD8^+^ T cells. FSC-area vs. FSC-height was used for doublet discrimination. A “dump” channel was then used to gate out dead cells (LIVE/DEAD fixable viability stain), CD14^+^ monocytes, and CD19^+^ B cells, and lymphocytes were selected by FSC vs. SSC. CD8^+^ T cells were subsequently selected on the basis of CD8 vs. CD3 staining, followed by the identification of proliferating (Ki67^+^) or activated (ICOS^+^) cells, and “effector CD8” cells as HLA-DR^+^CD38+. **b** Longitudinal empirical data, linear mixed models and estimated means (left, centre and right-hand panels respectively) for Ki67^+^ and ICOS^+^ CD8^+^ T cells, and HLA-DR^+^CD38^+^ effector CD8^+^ T cells (*P*-values: * <0.05, ** <0.01, *** <0.001, **** < 0.0001)

We further analysed the CD8+ T cell compartment for markers of proliferation and activation (see Fig. 5a for flow cytometry gating strategy). Proliferation was assessed by intracellular staining for Ki67, expressed in dividing and recently-divided cells [15]. Proliferation was seen to be highly cyclical, with the lowest proportion of Ki67^+^ cells consistently observed one week after chemotherapy (at Day 8) and the highest degree of proliferation one week after immunotherapy (at Day 15) for each cycle (Fig. 5b). The inducible co-stimulator molecule (ICOS), a member of the CD28 co-stimulator family, is expressed on activated T cells and is associated with antigen recognition [16, 17]. ICOS expression exhibited a similar pattern to Ki67, with a sharp decrease coinciding with blood samples collected one week following chemotherapy (Day 8) before returning to baseline levels (Fig. 5b). We also examined “effector” CD38^hi^HLA-DR^hi^ CD8+ T cells, which are activated in an antigen-specific manner as previously reported from studies of chronic viral infection, and are present at elevated levels in cancers including mesothelioma compared with healthy controls [18–22]. These cells are predominantly proliferating, and exhibit low expression of Bcl-2 (an anti-apoptotic protein downregulated following antigen-specific T cell activation) [19, 23]. Our raw data and subsequent linear mixed modelling data again show a marked cyclical pattern over each cycle of chemoimmunotherapy treatment, with the effector proportion of CD8+ T cells peaking at Day 1 of each cycle after dexamethasone prior to falling just below baseline levels at Day 8 following chemotherapy (Fig. 5b). None of the CD8+ T cell parameters described above showed a significant change over the 6 cycles of chemoimmunotherapy.

### CD4+ T cell proliferating, activated and regulatory populations

**Fig. 6:**
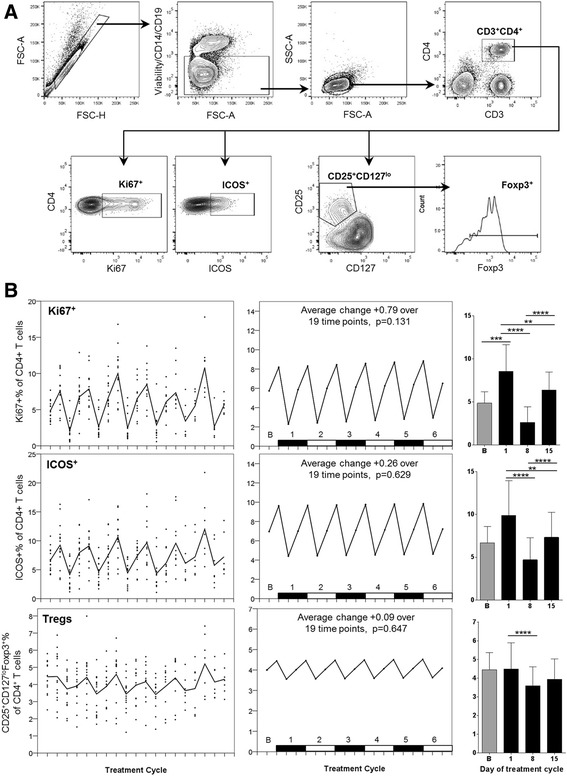
**a** Representative flow cytometry data, demonstrating the gating strategy used on PBMC for Treg identification and analysis. FSC-area vs. FSC-height was used for doublet discrimination. A “dump” channel was used to gate out dead cells (LIVE/DEAD fixable viability stain), CD14^+^ monocytes and CD19^+^ B cells, and lymphocytes were subsequently selected by FSC vs. SSC. CD4^+^ T cells were gated on the basis of CD4 vs. CD3 staining, then examined for expression of Ki67 and ICOS. Tregs were identified within the CD4^+^ T cell population as CD25^hi^CD127^lo^ and Foxp3^+^. **b** Longitudinal empirical data, linear mixed models and estimated means (left, centre and right-hand panels respectively) for Ki67+ and ICOS+ expression on CD4+ T cells, and the Treg proportion of CD4 cells (*P*-values: * <0.05, ** <0.01, *** <0.001, **** < 0.0001)

CD4+ T cells were also assessed by flow cytometry for overall proliferation (Ki67), activation (ICOS), and for the proportion of CD25^+^CD127^lo^Foxp3^+^ regulatory T cells (Tregs) present within the CD4+ T cell compartment, using the gating strategy described in Fig. 6a. Overall, CD4+ T cells exhibited a broadly similar profile for fluctuations in proliferation and activation as CD8+ T cells, with significant reductions in the proportion of both Ki67+ and ICOS+ cells at Day 8 of each cycle, one week following chemotherapy (Fig. 6b). Analysis of Tregs also revealed a cyclical pattern, albeit less pronounced, with Tregs decreasing by approximately 20% between Day 1 and Day 8 of each treatment cycle followed by a return to around baseline levels a week later (Fig. 6b). There was no change over the six treatment cycles in any CD4+ T cell populations.

**Fig. 7:**
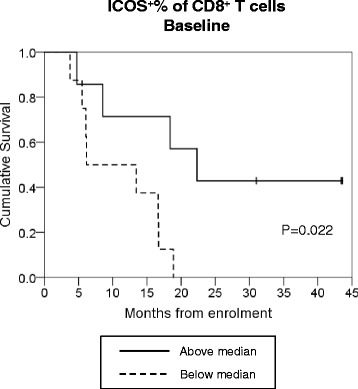
Overall survival data grouping patients above or below the median for the ICOS^+^% of CD8+ T cells at baseline. Statistical significance calculated using Mantel Cox log rank test. Solid and dotted lines show data above and below the median values, respectively

Flow cytometry data on T cell subset representation and expression of activational markers underwent exploratory analysis for correlation with OS, separately for each time point between baseline and Cycle 3 Day 8. For a full list of T cell parameters analysed, see Table 2. The majority of analyses showed no significant differences in OS above vs below the median, however those patients who had an ICOS+ % of CD8+ T cells above the median at baseline achieved better OS (Fig. 7). Both positive predictors of better OS in baseline samples, BDCA2+ DC% and ICOS + % of CD8+ T cells, were seen to correlate (*r* = 0.563, *p* = 0.029).

## Discussion

It is becoming increasingly important to identify immune biomarkers in patients treated with the variety of immunotherapies currently being developed; both to assist with clinical decision making and to help understand the immunological basis of response, or lack thereof. Given that activating anti-CD40 has strong preclinical and early clinical evidence of efficacy, we undertook this study of systemic immune parameters to establish the pattern of changes induced by this agent in the context of chemotherapy, and to undertake an exploratory analysis of any correlation between these changes and patient outcomes.

We recently published the results from a phase Ib clinical trial combining the anti-CD40 agonist antibody CP-870,893 with cisplatin/pemetrexed chemotherapy in patients with mesothelioma [9]. We demonstrated the combination was well tolerated, and had at least equivalent efficacy to chemotherapy alone. In an earlier study, Vonderheide et al. documented short-term changes in various immunological parameters immediately following CP-870,893 infusion on B cells in particular, and in our original report we described similar changes in B cell markers over 6 treatment cycles [4]. Here, we expand this longitudinal analysis of immune parameters to include data on dendritic cells and T cells, and show how, in the context of chemotherapy, CP-870,893 induces alterations in these cell populations.

Changes in B cells are one of the clearest immunopharmacodynamic indicators of CP-870,893 activity both in this and the previous study. This is not unexpected since B cells are the largest leukocyte subset that express the target of the drug, CD40. Notwithstanding this, the proposed mechanism of action of the drug is via agonistic activation of DC through the CD40 receptor [24, 25]. However, it remains to be clarified which DC subsets may be involved in anti-CD40 mediated tumour immunity.

BDCA-1+ (CD1c+) DC are the most numerous myeloid DC population, maturing into both tissue and lymph node DC that are potent activators of CD4+ T cells. BDCA-2+ (CD303+) plasmacytoid DC are present in the blood at similar concentrations to BDCA-1+ DC and are primarily lymphoid DC precursors; these play a key role in responding to viral infection through type 1 interferon release. BDCA-3+ (CD141+) myeloid DC are a smaller population, typically making up around 10% of total blood DC, and have a major role in cross-presentation of antigen to CD8+ T cells. See refs [26, 27], and for recent reviews on DC subtypes and nomenclature [26, 27]. We report that the proportion of the two major DC subsets, BDCA-1+ (CD1c^+^ myeloid) and BDCA-2+ (CD303^+^ plasmacytoid), decreased sharply as a proportion of total PMBC in response to dexamethasone treatment as previously described, but it was not apparent that anti-CD40 treatment itself (i.e. between Day 8 and Day 15) induced any changes away from baseline in either proportional presence of any DC subsets at the time points chosen for this study [14]. A previous study by Rϋter et al. reported that depletion from peripheral blood of CD11c^+^CD123^dim^ CD14^−^ DC was observed in the shorter term, although data were not shown [10].

It may be that direct binding to the CD40 molecule by CP-870,893 can modulate CD40 expression, but this was not observed - either because no such modulation occurred, or alternatively because it was simply not detected due to post-CP-870,893 blood collection being scheduled one week following drug administration, with any modulations occurring in the shorter term. Indeed, this was the case with data on B cell populations in previous work from this study [9]. Although expression of the MHC-class II molecule HLA-DR had a downward trend over 6 cycles of chemoimmunotherapy in all three DC subsets examined, this was only statistically significant in BDCA-1+ DC (*p* = 0.022) and we do not consider the magnitude of this decrease to be biologically relevant. A 2007 study by Hunter and colleagues reported that HLA-DR expression increased in response to CP-870,893 treatment of monocyte-derived DC in vitro over a 24 h time window. Similarly, whilst profound immediate and short term effects of CP-870,893 on B cells were identified, these parameters returned to baseline by day 8 after treatment [4]. Therefore it is possible that any increase was transient and not detected by us 7 days after treatment [11].

Weekly CP-870,893 monotherapy has been shown to reduce lymphocyte concentrations in peripheral blood to below 50% of baseline counts two days after treatment at MTD, followed by rebound to pretreatment levels, although this has not been observed for less frequent dosing schedules [4]. Across the longer term, however, our combination of cisplatin/pemetrexed and anti-CD40 gives similar outcomes to anti-CD40 monotherapy, with some variation between patients but generally no overall change in lymphocyte concentration observed [7, 10]. Ruter and colleagues also noted a bias toward depletion of CD4+ over CD8+ T cells and indeed our linear mixed modelling shows that over 6 cycles of chemotherapy this may be the case – particularly at Day 1 of each cycle, directly prior to administration of chemotherapy [10]. However our observation is also small in scale, and we are unable to say definitively whether anti-CD40 may be responsible. A high intrapatient variability in the proportional presence of Tregs was observed, but was generally modulated consistently within individual patients in response to treatment, no long term changes appeared to be uniformly induced with this 6-cycle treatment schedule in agreement with past studies [7, 10].

Our exploratory analyses of the relationship between immunological parameters and patient outcomes highlighted a potential relationship between either higher-than-median BDCA-2 + DC% of PBMC at baseline, or lower-than-median BDCA-3 + % at C2D8 and C3D8, and longer patient survival. Mechanistically, it is possible that a higher proportion of BDCA-2+ DC at baseline indicates a larger number of target cells available for the action of anti-CD40 therapy, potentially giving a useful prognostic indicator for those patients likely to achieve better responses. The seemingly beneficial link between a reduction in antigen cross-presenting BDCA-3+ DC and better OS, however, might seem contrary to expectation if an anti-tumour immune response is thought to be underlying the difference in survival. However, it may be that these BDCA-3+ are migrating out of the peripheral blood and into other tissues, potentially as a result of anti-CD40 inducing the maturation of these cells, and may therefore be indicative of those patients achieving better responses. Exploratory analyses in the T cell compartment revealed a correlation between OS and the ICOS + % of CD8+ T cells at baseline. The postulated mechanism of CP-870,893 action is to increase APC-mediated CD8+ T cell activation and it may be that those patients with higher-than-median CD8 activation prior to treatment have an immune landscape that is already predisposed toward this outcome; thus, addition of anti-CD40 is sufficient to tip the balance further in favour of a better anti-tumour response in those individuals. It is interesting to note that both parameters showing differences in OS at baseline, BDCA2+ DC and ICOS+ CD8 T cells, correlated in this sample set and may be worth further investigation. We acknowledge these data were obtained from a small number of patients and serve only as hypothesis-generating, however they may inform investigators in future clinical trials that include greater numbers of participants.

The interpretation of our results is complicated because each of the three administered agents have potential immune-modulating effects, and each was given at different stages of the 21-day treatment cycle. Nevertheless, this complexity is an emerging reality in human cancer immunotherapy, where single agents have promising but insufficient activity and where combinations are now under intense investigation. Firstly, characteristics of samples taken at Day 1 of each cycle (taken immediately before infusion of pemetrexed/cisplatin) were influenced by three doses of the glucocorticoid steroid dexamethasone, with a cumulative dose of 12 mg given in the 24 h leading up to chemotherapy. Dexamethasone is given prior to and for three to five days after pemetrexed/cisplatin for control of skin rash, emesis and inflammatory side effects, and has been shown to cause alterations in the majority of immune parameters examined in this paper; for example lymphodepletion and alteration of DC subsets [14]. Secondly, the effects of chemotherapy itself, given on Day 1 of each treatment cycle here and evident one week later at Day 8. The effects of cisplatin/pemetrexed are well known, particularly with respect to depletion of proliferating and activated cells as can be observed from our lymphocyte data, and may well alter the immunological background of patients in a manner that can affect the subsequent dose of anti-CD40. We have previously demonstrated a profound reduction in the proportion of Ki67+ CD8+, CD4+ and Treg as well as activated effector CD8+ T cells on day 8 after chemotherapy, which recovers to baseline prior to the next chemotherapy cycle [18]. Thirdly, the effects of the agonistic anti-CD40 antibody, administered on Day 8. As described above, pharmacodynamics of CP-870,893 with respect to parameters of the adaptive immune system are generally observed over a shorter time window (3–4 days) [4, 10]. It is therefore probable that the majority of direct effects of anti-CD40 have reset by the time the next blood sample is collected at Day 15, hence were not observed as part of the inter-cycle variation in our study.

An important message from this study is that investigators must undertake careful preliminary pharmacodynamic immunological studies when selecting time points for immunological biomarker investigation in patients who are undergoing concurrent chemoimmunotherapy. Although longitudinal samples from patients provide the opportunity to identify predictive biomarkers for response to combination therapy, it is more complicated to attribute changes in defined immunological parameters to individual drugs within this combination without the inclusion of control arms to the study. Ideally, control arms would monitor immunological parameters in individual agents, however this is not always practical or feasible with small signal-finding studies and phase Ib studies. Consistency in timing of immunological sampling is paramount, with the potential for alteration in immunological parameters by supportive care medications and chemotherapy in addition to immunotherapies. A pre-corticosteroid baseline is also essential; some chemotherapy regimens have an absolute requirement for corticosteroid premedication which cannot be modified. Furthermore, in many clinical trials there is an allowable window for blood sampling which may include +/− one to two days which may risk adding to the complexity of result interpretation. Statistical techniques such as the linear mixed modelling used in this study can enhance the clarity of interpretation of complex, cyclical data and facilitate an understanding of change over time. However, only the availability of a control group receiving chemotherapy and supportive care alone will allow the contributions of immunotherapy to biomarker changes to be definitively evaluated.

## Conclusions

This study did not find any longitudinal changes in the profile of dendritic cells or T cells associated with this treatment, and conclude that B cells remain the best indicator of CP-870,893 pharmacodynamic activity across multiple cycles of treatment in combination with pemetrexed and cisplatin. Careful consideration of scheduling, consistency, and premedications is required in interpreted immunological change in chemoimmunotherapy trials. The availability of a control group is likely critical to robust interpretation of any potential biomarkers.

## Abbreviations

APC: Antigen presenting cell
DC: Dendritic cell
ICOS: Inducible co-stimulator
MTD: Maximum tolerated dose
OS: Overall survival
PBMC: Peripheral blood mononuclear cell
Treg: Regulatory T cell
